# L-lysine as adjunctive treatment in patients with schizophrenia: a single-blinded, randomized, cross-over pilot study

**DOI:** 10.1186/1741-7015-9-40

**Published:** 2011-04-18

**Authors:** Caroline Wass, Daniel Klamer, Evangelos Katsarogiannis, Erik Pålsson, Lennart Svensson, Kim Fejgin, Inga-Britt Bogren, Jörgen A Engel, Birgitta Rembeck

**Affiliations:** 1 Department of Pharmacology, Institute of Neuroscience and Physiology, The Sahlgrenska Academy at University of Gothenburg, PO Box 431, 405 30 Gothenburg, Sweden; 2 Psychiatric Clinic/PVV, Sahlgrenska University Hospital, Topasgatan 2, 421 48 V:a Frölunda, Sweden; 3 Schizophrenia Program, Centre for Addiction and Mental Health, Toronto, Canada; 4 Department of Psychiatry, Division of Addiction Psychiatry, University of Toronto, Toronto, Canada

## Abstract

**Background:**

Accumulating evidence suggests that the brain's nitric oxide (NO) signalling system may be involved in the pathophysiology of schizophrenia and could thus constitute a novel treatment target. The study was designed to investigate the benefit of L-lysine, an amino acid that interferes with NO production, as an add-on treatment for schizophrenia.

**Methods:**

L-lysine, 6 g/day, was administered to 10 patients with schizophrenia as an adjunctive to their conventional antipsychotic medication. The study was designed as a single-blinded, cross-over study where patients were randomly assigned to initial treatment with either L-lysine or placebo and screened at baseline, after four weeks when treatment was crossed over, and after eight weeks.

**Results:**

L-lysine treatment caused a significant increase in blood concentration of L-lysine and was well tolerated. A significant decrease in positive symptom severity, measured by the Positive And Negative Syndrome Scale (PANSS), was detected. A certain decrease in score was also observed during placebo treatment and the effects on PANSS could not unequivocally be assigned to the L-lysine treatment. Furthermore, performance on the Wisconsin Card Sorting Test was significantly improved compared to baseline, an effect probably biased by training. Subjective reports from three of the patients indicated decreased symptom severity and enhanced cognitive functioning.

**Conclusions:**

Four-week L-lysine treatment of 6 g/day caused a significant increase in blood concentration of L-lysine that was well tolerated. Patients showed a significant decrease in positive symptoms as assessed by PANSS in addition to self-reported symptom improvement by three patients. The NO-signalling pathway is an interesting, potentially new treatment target for schizophrenia; however, the effects of L-lysine need further evaluation to decide the amino acid's potentially beneficial effects on symptom severity in schizophrenia.

**Trial registration:**

NCT00996242

## Background

Schizophrenia is a severely debilitating brain disorder that poses a serious healthcare problem worldwide. Available antipsychotics show efficacy in alleviating psychotic symptoms. However, negative symptoms and the cognitive deficits are to a large extent resistant to antipsychotic treatment [1,2]. Thus, there is a need to find new treatment strategies to improve the treatment of these symptoms and deficits. One such candidate target for novel treatments may be the nitric oxide (NO) signalling system of the brain. As such, translational evidence for this contention can be derived from the observations that methylene blue, which blocks NO-dependent soluble guanylate cyclase-mediated intracellular signalling, was shown to exert therapeutic effects as an adjuvant to established antipsychotics in the treatment for schizophrenia in humans [3]. In addition, a more recent study showed that methylene blue attenuated psychotomimetic-, that is, phencyclidine (PCP), induced behavioural alterations in mice [4]. Despite that the principle mechanism of action of PCP is glutamatergic N-methyl D-aspartate (NMDA) receptor antagonism, PCP has secondary effects on several other neurotransmitter systems (see, for example, [5]) as well as on NO-signalling [6]. Furthermore, the inducible NO synthase inhibitor, minocycline, was recently suggested to have beneficial effects as an add-on treatment in patients with schizophrenia [7,8]. Thus, accumulating evidence indicates that alterations in NO function may be involved in the pathophysiology of schizophrenia and these original findings motivate further investigations of the potential utility of NO modulation as a novel pharmacological treatment rationale for schizophrenia (for review, see [9]).

### Preclinical findings supporting a "NO dysregulation hypothesis for schizophrenia"

To better understand the underlying pathophysiology of schizophrenia, several methods have been developed to model schizophrenia in humans and experimental animals. To this end, pharmacological challenge with PCP has been shown to produce a psychotic condition in humans that includes all major symptoms of schizophrenia. Thus, the "PCP model of schizophrenia" has proved to be an important tool for increasing our understanding of the disorder and is considered to have significant heuristic value in the development of novel therapeutic treatment strategies [10].

Interestingly, our preclinical studies have shown that pre-treatment with NO synthase (NOS) inhibitors effectively block the disruptive effect of PCP on behaviours involving several cognitive domains such as pre-attentive information processing, non-associative learning, selective attention, cognitive flexibility, working and long-term memory, as well as deficits in social interaction in rodents [11-20]. These observations suggest that inhibition of NOS is able to counteract very complex behavioural effects of PCP.

### Inhibition of NO production by L-lysine: A new treatment option for patients with schizophrenia?

NO is produced from the amino acid L-arginine and molecular oxygen in a chemical reaction catalyzed by NOS. Interestingly, the essential amino acids L-lysine and L-arginine share a highly specific membrane bound transport system, the cationic amino acid transporter [21]. This transport system seems critical in mediating the influx of L-arginine across the blood-brain barrier [22]. Furthermore, *in vitro *studies have shown that saturation of the transporter with L-lysine inhibits transport of L-arginine, depletes intra-cellular stores of L-arginine [23], and reduces NO production [24].

As noted above, our previous findings suggest that the schizophrenia-like behavioural effects of PCP in experimental animals can be ameliorated by pre-treatment with a NOS inhibitor. In analogy it may be speculated that inhibition of L-arginine transport by L-lysine treatment would decrease NO levels and, thereby, attenuate PCP-induced behavioural effects. In support of this argument we recently showed that sub-chronic pre-treatment with L-lysine ameliorates PCP-induced disruption of prepulse inhibition, a measure of pre-attentive information processing, in mice in a dose-related manner without affecting basal prepulse inhibition [25]. Moreover, using a NO selective microsensor and *in vivo *voltammetry in awake freely moving rats, we recently found that acute L-lysine administration decreased NO levels in the rat prefrontal cortex [26]. A competitive antagonism of L-arginine transport across the blood-brain barrier and a depletion of L-arginine supply for NO synthesis may explain these findings. A relative lack of substrate for NO production would prevent a hypothesized PCP-induced increase in NO levels and thus the disruptive effect of PCP on cognitive function.

The aim of the present study was to investigate the relevance of these contentions in humans. To this end, the effect of adjunctive L-lysine treatment on symptom severity and cognition was studied in patients with schizophrenia. Ten patients with a diagnosis of schizophrenia were treated with L-lysine, 6 grams daily, or placebo for four weeks in a cross-over design. Outcome measures were assessed at baseline, after four weeks and after eight weeks.

## Methods

### Subjects

Eleven well-defined psychiatric outpatients were recruited from the following three clinics: Nå-Ut Teamet, Psykosteamet Järntorget, and Psykosvård i Väster, the Sahlgrenska University Hospital, Gothenburg, Sweden. Inclusion criteria consisted of a DSM-IV diagnosis of schizophrenia (F295.xx) and that the patient had been in a stable phase of illness (that is, no psychotic episode) during the last two months preceding study participation. Furthermore, all subjects were required to be on a stable dose of antipsychotic medication, in addition to any other prescribed medication, for at least three months before the start of the study (for specification of prescribed antipsychotic drugs, see Table 1). No major medical or neurological conditions or other psychiatric diagnosis (besides DSM-IV, F295.xx) were allowed as assessed by a comprehensive medical history and a medical examination. Furthermore, all subjects had to show normal admission laboratory tests and vital signs. Substance abuse, apart from smoking, was grounds for exclusion. The study was approved by the Swedish Medical Products Agency and the Internal Review Board at the University of Gothenburg, Sweden. One participant (subject number 9) terminated participation after four weeks of treatment due to non-compliance with the drug administration procedure, thus resulting in a total of 10 patients completing the study.

**Table 1:** Demographic data from all patients participating in the study.

Case	Age (years)	Sex	Subtype (DSM-IV)	Duration of illness (years)	Antipsychotic treatment, dosage
1	56	M	Schizophrenia, undifferentiated	9	Risperdal consta 25 mg/2w
2	23	F	Schizophrenia, undifferentiated	3	Leponex 700 mg/day,
3	29	M	Schizophrenia, unspecified	8	Risperdal 2 mg/day, Abilify 25 mg/day
4	45	M	Schizophrenia, paranoid	6	NA
5	35	M	Schizophrenia, unspecified	11	Leponex 200 mg/day, Abilify 15 mg/day
6	54	M	Schizophrenia, undifferentiated	29	Zyprexa 15 mg/day
7	51	M	Schizophrenia, paranoid	23	Leponex 100 mg/day, Abilify 10 mg/day
8	56	M	Schizophrenia, undifferentiated	> 15	Zyprexa 10 mg/day, Cisordinol 140 mg inj 1/4w
9	-	-	-	-	-
10	39	F	Schizophrenia, paranoid	8	Risperdal 4 mg/day
11	44	M	Schizophrenia, unspecified	26	Leponex 800 mg/day, Abilify 10 mg/day

Case 9 dropped out due to difficulties ingesting the study medicine.

### Demographic data

The demographic data of the patients enrolled in the present study are shown in Table 1.

### Study design

The study was designed as a single-blinded, placebo-controlled, eight-week cross-over study. A written consent was signed before patients entered the study and were randomly assigned to initial treatment with either L-lysine dissolved in a soft drink or a soft drink only (the placebo) and screened for the outcome measures at baseline, after 28 days of treatment when treatment was crossed over, and after 56 days when treatment was terminated.

### Study compound

L-lysine is a basic amino acid (that is, it carries a positive net charge at physiological pH) with a high nutritional value. It is readily absorbed from the intestine and metabolized by the liver. High levels of L-lysine are found in muscle, and increased intake of L-lysine is more readily distributed to this compartment than to, for example, blood. L-lysine supplementation has been tried as treatment for, for example, recurrent herpes simplex infection [27] and osteoporosis [28]. It is an essential amino acid and has very good oral bioavailability [29] and brain penetration [22]. L-lysine monohydrochloride is soluble in water and has a mild salty flavour. In the present study the compound was mixed with juice or a soft drink and ingested once a day. The dose of L-lysine was set to 6 g/day, a dose above dietary levels (dietary intake of L-lysine rich foods may reach 3 g/day) but within known safety limits [30]. L-lysine monohydrochloride was synthesized by Apoteket Produktion & Laboratorier, APL Stockholm.

### Safety aspects

The only reported adverse effects of L-lysine treatment in humans are transient gastrointestinal problems in a few subjects. Based on published studies, a long-term addition of 6 g of L-lysine to the daily diet should be safe [30]. In short-term human studies doses as high as 40 g/day have been tested and again the only reported adverse effects were abdominal cramps and transient diarrhoea that resolved as the dose was decreased [31,32]. Toxicological studies in rats indicate that the lethal dose 50 (LD_50_) of intravenous L-lysine is 4 mg/kg [33]. No lethal dosage could be reached by oral administration. Long-term studies on L-lysine administration (up to two years) have not revealed any adverse effects [30].

### Clinical assessments

Patients were screened for outcome measures at baseline, after four weeks, and after eight weeks. Symptom severity and functional outcome was assessed with the PANSS, and the UKU-Scale for monitoring side effects of psychiatric medication. Measures of physical status included blood sample analysis for L-lysine concentration and amino acid composition, blood pressure, pregnancy test and weight, all obtained by a trained research psychiatrist. Tests of cognitive performance included the Ray Auditory Verbal Learning Test (RAVLT), Continuous Performance Test (CPT; vigilance and attention), Trail Making Test A and B (speed of processing), Letter and Number Span test (working memory) and Wisconsin Card Sorting Test (WCST; working memory and executive functioning). All tests were carried out by a trained behavioural scientist.

### Amino-acid measures

Blood samples (4 ml) for assessment of essential amino acid profiling were obtained at baseline after L-lysine treatment, and after placebo treatment. The blood samples were sent to the Laboratory for Clinical Chemistry, the Sahlgrenska University Hospital, Gothenburg, Sweden, for analysis using a ninhydrin reagent for photometric determination of amino acids (for procedure, see [34]).

### Statistics

Statistical analysis was performed by one- or two-way ANOVA when appropriate and multiple testing was subjected to Bonferroni correction with the significance level set to 0.05. Linear regression analysis was also performed to test if blood level of L-lysine concentration could predict functional outcome of any of the outcome measures collected.

## Results

### L-lysine treatment significantly increased blood concentration of L-lysine without inducing adverse side effects

Analysis of L-lysine concentration in blood showed a significant effect of treatment such that concentration increased after L-lysine treatment (6 g/day for four weeks) compared to baseline and placebo levels (F(2,14) = 7.84, *P *< 0.05, Table 2), in 8 out of the 10 patients. Two patients showed no change in L-lysine concentration and were treated as non-responders and included in an "Intention to Treat" group (n = 10), which was analyzed in parallel with the responders (n = 8) in the statistical analysis. Furthermore, the L-lysine treatment was not found to induce any adverse side effects including extrapyramidal effects. Neither was L-lysine treatment found to significantly alter blood concentration of any of the other amino acids analysed, such as citrulline, arginine, proline, glutamate and alanine.

**Table 2:** Results before, after four weeks of L-lysine treatment, and after four weeks of placebo treatment.

Outcome measure	Baseline	Post L-lysine	Post placebo	F value	*P-*value*
L-lysine blood conc (μmol/l)	196 ± 14 (201 ± 15)	401 ± 74 (363 ± 64)	195 ± 13 (195 ± 10)	7.84 (6.57)	0.024 (0.027)

**Symptom severity scales**					

PANSS					
Positive symptoms	14.6 ± 0.7 (16.0 ± 1.1)	11.9 ± 0.6 (13.5 ± 1.3)	11.6 ± 0.8 (13.4 ± 1.4)	13.11 (12.49)	0.001 (0.000)
Negative symptoms	18.4 ± 2.4 (19.7 ± 2.2)	17.4 ± 2.2 (19.4 ± 2.2)	17.3 ± 2.4 (18.8 ± 2.2)	1.53 (0.69)	0.25 (0.51)
General symptoms	31.3 ± 2.5 (33.5 ± 2.5)	29.5 ± 1.9 (32.3 ± 2.4)	29.1 ± 2.0 (30.9 ± 2.0)	2.14 (2.84)	0.16 (0.09)
Total	64.3 ± 4.2 (69.2 ± 4.7)	58.8 ± 4.0 (65.2 ± 5.4)	58.0 ± 4.5 (63.1 ± 5.0)	13.36 (8.59)	0.001 (0.002)

GAF	43.8 ± 1.8 (42.0 ± 1.9)	46.9 ± 1.6 (45.8 ± 1.6)	44.1 ± 2.3 (42.8 ± 2.1)	4.29 (7.34)	0.035 (0.005)

CGI	3.63 ± 0.18 (3.80 ± 0.20)	3.63 ± 0.18 (3.80 ± 0.20)	3.50 ± 0.27 (3.70 ± 0.26)	1.00 (1.00)	0.39 (0.39)

**Depression scale**					

MADRS	8.50 ± 2.09 (9.10 ± 1.74)	7.50 ± 1.90 (7.90 ± 1.57)	8.38 ± 1.74 (8.40 ± 1.38)	0.54 (0.89)	0.59 (0.43)

**Cognitive tests**					

RAVLT	45.9 ± 4.3 (43.5 ± 4.2)	46.0 ± 4.6 (43.3 ± 4.4)	48.8 ± 3.5 (45.2 ± 4.1)	0.79 (0.48)	0.47 (0.62)

CPT	0.76 ± 0.16 (0.67 ± 0.13)	0.81 ± 0.13 (0.76 ± 0.12)	0.86 ± 0.13 (0.71 ± 0.14)	0.25 (0.21)	0.77 (0.81)

Trail Making Test A	33.8 ± 1.7 (39.2 ± 5.9)	31.3 ± 2.5 (39.9 ± 8.9)	35.3 ± 4.5 (40.8 ± 6.3)	0.44 (0.07)	0.58 (0.93)

Trail Making Test B	77.4 ± 7.2 (134 ± 56)	70.0 ± 5.2 (119 ± 48)	71.3 ± 6.4 (124 ± 50)	0.40 (1.61)	0.68 (0.24)

Letter and number span	8.13 ± 0.90 (7.00 ± 1.03)	9.00 ± 0.66 (8.10 ± 0.82)	9.13 ± 0.92 (8.50 ± 0.95)	0.74 (1.92)	0.50 (0.18)

Wisconsin Card Sorting					
Number of errors	33.9 ± 10.0 (31.9 ± 9.0)	21.0 ± 7.2 (19.7 ± 6.5)	24.4 ± 10.1 (23.2 ± 9.0)	4.91 (5.45)	0.024 (0.016)
Perseverant errors	20.4 ± 7.6 (19.0 ± 6.8)	11.5 ± 5.0 (10.7 ± 4.4)	14.8 ± 8.1 (13.4 ± 7.3)	4.46 (5.00)	0.032 (0.021)
Perseverant answers	23.6 ± 9.6 (21.9 ± 8.7)	13.1 ± 6.6 (21.1 ± 5.9)	17.1 ± 10.3 (15.6 ± 9.2)	4.77 (5.20)	0.026 (0.018)

Mean values outside parenthesis refers to analysis including individuals who did display an increase in blood levels of L-lysine during the L-lysine treatment period (n = 8). Mean values within parenthesis refer to statistical analysis performed on all individuals, that is, also those not displaying increased blood levels of L-lysine during the L-lysine treatment period (total n = 10). *Corrected by the Huynh-Feldt procedure as required.

### Positive symptom scores were significantly improved during treatment

Symptom severity as measured by the positive symptoms sub-scale of PANSS showed a significant decrease (F(2,14) = 13.11, *P *< 0.001). This effect was mainly due to a significant change in scores for the sub-scales assessing delusions and suspiciousness/persecution. It should be noted that the patients tended to improve their performance on the positive symptoms PANSS measure regardless of treatment, an effect that may be explained by the increased attention the patients received simply by taking part in the study. Since such an effect would jeopardize the interpretation of the results, measures were taken to control for this effect by applying a two-way ANOVA to the data analyzing the interaction between treatment order (L-lysine treatment during first or second testing period) and test session (Baseline, Test 1 and Test 2). The rationale behind this approach was that given a biologically significant effect of L-lysine, a major part of the improvement in the testing score should be attributed to the L-lysine treatment period, and less to the placebo period and this effect should be detected in the interaction analysis. Albeit close to a significant interaction (F(2,12) = 3.08, *P *= 0.08), this analysis did not reach statistical significance and consequently it could not be excluded that a significant part of the effects obtained on the PANSS score was caused by factors not related to L-lysine treatment.

### Problem solving capacity was significantly improved during treatment

Problem solving capacity and cognitive flexibility, as assessed by the WCST, was significantly improved following treatment. This effect was statistically significant measured as decrease in the number of errors on the WCST (F(2,14) = 4.91, *P *< 0.05, Table 2), measured as perseverant errors (F(2,14) = 4.46, *P *< 0.05), and measured as perseverant answers (F(2,14) = 4.77, *P *< 0.05). A similar tendency of the patients to improve their WCST performance regardless of treatment was observed. Statistical analysis did not show a significant interaction between treatment order and test session and consequently the effects obtained were caused by factors not related to L-lysine treatment.

The RAVLT, CPT-IP, TRAIL MAKING A and B, and Letter and Number Span test outcome scores were not significantly affected by treatment.

Besides the outcome measures, patients self-reported on their condition, most commonly once a week when receiving the study drug. As such, no patient reported any adverse side-effects or worsening of symptoms. Interestingly though, three patients self-reported improvements of their symptoms. Two patients reported a decrease in positive symptoms, such as attenuated auditory hallucinations (*i.e*. hearing voices), an improvement that disappeared after trial termination. One of these patients also experienced improved attention following L-lysine treatment and one other patient reported increased mental stability and memory capacity following L-lysine treatment, an effect that remained several weeks after trial termination.

## Discussion

Evidence was obtained suggesting that 6 g/day of adjunctive L-lysine treatment is a sufficient dose for increasing blood L-lysine levels above the nutritional, naturally occurring levels, without inducing adverse side-effects. In addition, there was a significant decrease in the positive PANSS scores and WCST over the whole study period. These two measures represent different functional abilities in the individual; PANSS is used to measure psychosis severity and WCST cognitive functionality. Thus, evidence was obtained for symptom improvement in both of these domains; however, accounts for placebo effects, such as training on WCST, cannot be excluded.

Given the limited number of patients enrolled in the study, difficulties arise as to the possibilities of making reliable conclusions about the efficacy of L-lysine treatment. However, it should be noted that despite the low number of participants statistically significant effects of treatment on outcome measures were in fact obtained. Furthermore, albeit the relatively small changes in PANSS and WCST scores observed, statistical analysis showed fairly high effect sizes and observed powers for these changes (positive PANSS (n = 10): effect size (η) = 0.76, observed power (1-β) = 0.99; WCST number of errors (n = 10): effect size (η) = 0.64, observed power (1-β) = 0.77) indicating that they were of significant magnitude and detectable also in small sample sizes. Needless to say, it is also possible that a higher dose of L-lysine or a longer treatment period may have provided more clear-cut results. The reason that two participants (one male and one female) did not get an elevation in their blood concentration of L-lysine is unknown. In order to assess adherence to the L-lysine treatment the L-lysine containers were carefully labelled with dates and empty containers were collected weekly. However, there was no control for actual intake of the study medication beyond the assessed blood L-lysine concentrations. Metabolic individual differences, due to *i.a*. intense physical activity or gender, or simply non-adherence to L-lysine treatment, cannot be ruled out as one of the contributing factors to these findings.

Cautions should be taken when interpreting the data. Both the PANSS and the WCST scores seemed to improve over repeated testing, and consequently this placebo and/or training effect could explain the effects obtained. The study design presently used did not provide a satisfactory control for such effects, but the statistical measures, that is, analysis of interaction between treatment order and test session on outcome measure, that were taken to address this issue confirmed the possibility of placebo and training effects. Indeed, the WCST has been shown to be sensitive to training effects (see, for example, [35]) and should be used with care. Ideally, a test less sensitive to training and with higher test-retest reliability may be advisable for future studies, as well as a pre-testing cognitive training session (before the baseline assessment) in order to control for training artefacts.

The fact that the patients enrolled in the study were in a stable phase of their illness and were all treated with atypical antipsychotics may also have made it more difficult to detect beneficial effects of L-lysine treatment on cognitive functioning and symptom severity. In this respect, special attention may be paid to the self-reported improvements of positive symptoms, attention and memory capacity by three of the patients, since these potential effects of the treatment would be important but unfortunately could not be addressed by statistical analysis. It should also be noted that the patient who experienced improved memory capacity continuously trained his memory using a computerized training program.

There is an ongoing debate in the literature as to how to disentangle training effects of repeated cognitive testing, from "real" drug effects, as well as how to establish consensus regarding a reliable cognitive test battery [36-38]. The cognitive tests chosen for the purpose of the present study are some of the most frequently applied in research and several of them have been shown to be resistant to training effects [39,40]. The initiatives taken to pharmacologically enhance cognition are plentiful; however, the results of such studies are diverse and few studies have been able to demonstrate replicable cognitive enhancement in patients with schizophrenia [38]. One contributing factor to these shortcomings, may be that schizophrenia encompasses abnormal progressive loss in brain volume [41], possibly counteracting any cognitive enhancing potential. However, cognitive enhancing therapy was recently demonstrated to counteract gray matter loss in patients with schizophrenia [42].

Cognition is a highly specific and complex phenomenon that develops from early life into adulthood, depending on both biological pre-disposition as well as environmental input [36]. Perhaps the lack of development of cognitive enhancing agents is due to an overly optimistic belief in cognitive enhancement being viable without concurrent cognitive training. The plasticity of the brain is a complex, time- and stimulus-dependent process by which the strengthening of synapses has to be modulated in order for learning to occur [43]. Thus, it may in fact not be possible to find improvements in cognition by solely adding cognitive enhancing compounds without simultaneous cognitive training. Therefore, it is interesting that L-lysine treatment in combination with training was perceived to improve memory capacity in one patient. Moreover, adjunctive L-lysine treatment needs to be investigated in a larger placebo-controlled, double-blinded study further limiting possible training effects.

Taken together, preclinical and clinical studies have provided evidence for the involvement of a brain NO imbalance in schizophrenia. Furthermore, preliminary *in vivo *studies in rats have indicated that NO production is decreased by L-lysine [26]. Consequently, L-lysine may have a beneficial potential in the treatment of the disorder. In addition, targeting the NO pathway may represent a new therapeutic approach that is pharmacologically fundamentally different from that of the traditional antipsychotics and, hence, may be beneficial in targeting symptoms that are currently treatment resistant.

## Conclusions

To our knowledge, the present study is the first to investigate L-lysine treatment as a potential new add-on treatment for patients with schizophrenia, and as a novel means of targeting the NO pathway in order to treat a psychiatric disorder.

## Competing interests

The authors declare that they have no competing interests.

## Authors' contributions

This study is to the best of our knowledge the first investigation to ever evaluate the effects of L-lysine treatment in patients with schizophrenia. CW wrote the study protocol, coordinated the study, carried out cognitive tests and wrote the first version of the manuscript. DK planned and initiated the study, analyzed and interpreted data as well as co-authored the manuscript. EK was the physician carrying out the clinical assessments including PANSS, MADRAS and CGI together with responsible senior specialist in psychiatry BR; they have both co-authored the paper. KF and EP did the ethical application, analyzed and interpreted the data as well as wrote the report. In addition, EP planned and made necessary pharmacological and pharmacokinetic research to determine the dosing of L-lysine in the present investigation. LS wrote and managed the application to the Swedish Medical Products Agency and carried out all the statistical analyses, and co-authored the manuscript. I-BB carried out cognitive testing, supervised CW and helped interpret the results. JAE provided financial means as the majority of grants were sought by him, as a professor in pharmacology and an M.D. JAE provided medical expertise in the planning and managing of the study as well as during analysis and interpretation of the data. JAE also co-authored the report. All authors read and approved the final manuscript.

## Pre-publication history

The pre-publication history for this paper can be accessed here:

http://www.biomedcentral.com/1741-7015/9/40/prepub
